# The State of Cardiac Rehabilitation in Saudi Arabia: Barriers, Facilitators, and Policy Implications

**DOI:** 10.7759/cureus.48279

**Published:** 2023-11-04

**Authors:** Tasneem Adam, Abdullah I Al Sharif, Taghreed Saeed M Alamri, Rawan Ahmad O Al-Nashri, Alaa Ibrahim M Alluwimi, Amani Yosef Samkri, Mohammed Abdullah Alharthi, Ahmed Yahya Moafa, Nawaf A Alsaadi, Abdullah Mahdi S Alraimi, Reham Hassan M Alquzi

**Affiliations:** 1 Medical Affairs, College of Applied Medical Sciences, King Saud Medical City, Riyadh, SAU; 2 Healthcare Planning and Development, Ministry of Health, Kingdom of Saudi Arabia, Riyadh, SAU; 3 College of Medicine, Alfaisal University, Riyadh, SAU; 4 General Practice, Primary Healthcare Center, Ministry of Health, Medina, SAU; 5 General Practice, Primary Healthcare Center, General Directorate of Health Affairs, Aseer, SAU; 6 Internal Medicine, Dar As Salama Medical Hospital, Al-Khobar, SAU; 7 General Practice, Al Aziziah Primary Health Care Center, Ministry of Health, Makkah, SAU; 8 General Practice, Royal Saudi Land Forces, Ministry of Defence, Riyadh, SAU; 9 Internal Medicine, Jazan University, Jazan, SAU; 10 Medical Affairs, College of Medicine, King Saud Medical City, Riyadh, SAU; 11 Faculty of Medicine, Thamar University, Dhamar, YEM; 12 Cardiology, Umm Al-Qura University, Makkah, SAU

**Keywords:** lifestyle change, preventive cardiology, cardiac rehabilitation, public health, saudi arabia, cardiovascular disease

## Abstract

Cardiovascular disease (CVD) is a critical public health issue in Saudi Arabia, where it is the leading cause of death. The economic burden of CVD in the country is expected to triple by 2035, reaching $9.8 billion. This paper provides an overview of CVD in Saudi Arabia and its risk factors, impact on healthcare, and effects on patients' quality of life. The review emphasizes the potential of cardiac rehabilitation (CR) programs in addressing the CVD epidemic. CR programs have been shown to reduce morbidity, mortality, and hospital readmissions while improving patients' cardiovascular health and overall well-being. However, these programs are underutilized and inaccessible in Saudi Arabia. The paper highlights the urgent need for CR programs in the country and suggests key strategies for implementation. These include increasing patient referrals, tailoring programs to individual needs, enhancing patient education, and making CR accessible through home-based options. Fostering multidisciplinary collaboration and developing tailored guidelines for Arab countries can further enhance the impact of CR programs. In conclusion, this review underscores the vital importance of comprehensive CR programs in Saudi Arabia to combat the rising CVD burden, improve patient quality of life, and align with the goals of the Saudi 2030 Vision for a healthier society.

## Introduction and background

Cardiovascular disease (CVD) is a broad term used to encompass a group of diseases that impact the heart and blood vessels, including conditions like coronary heart disease, cerebrovascular disease, and rheumatic heart disease, among others. When these diseases affect the vessels in the coronary artery or when a thrombus occludes vital organs such as the heart, brain, kidneys, and eyes, it can result in disability for these organs and their functions. This disability can manifest in various ways, including blindness, diabetes mellitus, stroke, psychotic disorders, an increase in mortality rates, accelerated aging, and a reduction in daily activity [1]. CVD stands as the leading cause of death worldwide, claiming an estimated 17.9 million lives each year [2].

CVD is the leading cause of mortality in Saudi Arabia, accounting for approximately 45.7% of all deaths [3]. Currently, in Saudi Arabia, the annual mortality rate of CVD stands at 294 per 100,000 individuals, while an additional 3,702 per 100,000 individuals suffer from non-fatal CVD [4]. CVD is a prominent health concern with a noteworthy prevalence that is consistently increasing. By the year 2035, it is projected that the prevalence of CVD will rise, affecting 479,500 individuals in the country. Furthermore, the financial burden associated with CVD, encompassing both direct and indirect costs, is anticipated to triple, reaching a staggering amount of US$9.8 billion [5]. 

Moreover, cardiac diseases have a major impact on patients' quality of life from various perspectives. Firstly, the reduction in cardiopulmonary function leads to activity limitations and decreased exercise capacity, resulting in a decline in self-care behaviors and independence. This, in turn, leads to frequent emergency visits or hospitalizations due to the frequent exacerbation of their condition [6]. Secondly, there is a deterioration in psychosocial well-being, as many patients may develop depression, anxiety, and insomnia [7]. Furthermore, studies have shown that individuals with CVDs often require time off work for recovery from illness or surgery. Additionally, there is a higher occurrence of presenteeism and absenteeism among individuals at moderate to high risk of coronary artery disease and ischemic stroke [8,9].

Cardiac rehabilitation (CR) programs are comprehensive initiatives aimed at secondary prevention, offering a range of services and long-term medical interventions. These encompass medical evaluations, prescribed exercise regimens, modifications to cardiac risk factors, educational sessions, and counseling [10]. The primary goal is to enhance the overall physical, mental, and social well-being of patients while reducing the risk of future heart problems [11,12]. Additionally, CR programs have been shown to reduce morbidity and mortality rates, as well as the number of hospital readmissions [13]. In a Saudi Arabian study involving 82 post-coronary artery bypass graft surgery patients, a randomized controlled trial was conducted to evaluate the efficacy of home-based cardiac rehabilitation compared to outpatient-based cardiac rehabilitation and usual care. The results showed that home-based cardiac rehabilitation was just as effective as outpatient-based cardiac rehabilitation, and it exhibited a superior ability to sustain improvements beyond the intervention period [14]. Another study was conducted to investigate the impact of different doses of CR programs on major adverse cardiovascular events. The study found a direct association between attendance at CR sessions and a reduction in the risk of major adverse cardiovascular events [15]. 

Based on this evidence, the American Heart Association has endorsed CR programs to improve cardiovascular health for individuals who have experienced heart attacks, heart failure, angioplasty, or heart surgery [16,17]. However, CR programs in Saudi Arabia are not universally available or accessible to all patients. The present review aims to examine the need for a CR program in Saudi Arabia. In this review, we provide a detailed summary of various factors, such as the prevalence of CVD in Saudi Arabia, the current status of CR programs, the effectiveness of such programs in improving outcomes, and the potential benefits and challenges associated with implementing and promoting CR in the country.

## Review

Public health burden of CVD in Saudi Arabia

Prevalence and Incidence of CVD In Saudi Arabia

Throughout history, infectious diseases and malnutrition have been recognized as the predominant contributors to mortality. However, over the last century, CVD has emerged as the preeminent global cause of death, accounting for 31% of all deaths [18]. This holds true not only for industrialized nations in Western Europe, North America, and East Asia but also for the overwhelming majority of developing countries [19]. 

In Saudi Arabia, the rates of CHD were lower compared to Egypt, with percentages of 5.5% and 8%, respectively. It is important to note that the Saudi report is somewhat outdated and may not fully reflect the current situation [20]. Furthermore, there have been no recent studies reporting the prevalence of CVD in Saudi Arabia or its regions. However, a study conducted in Saudi Arabia aimed to investigate the prevalence of CVDs among patients with type 2 diabetes mellitus [21]. The study reported that out of 883 patients, 158 had established CVD (18%). Additionally, while the prevalence of different subtypes of CVDs was relatively lower in the Saudi Arabian population compared to the global sample, it's worth noting that coronary heart disease remained the most prevalent subtype of CVD in both Saudi Arabia and the global population [21]. The incidence of CVDs in Saudi Arabia is relatively high, with an estimated 32,200 new cases of heart failure (HF) diagnosed yearly [22]. In 2010, the MOH reported that CVDs accounted for 42 percent of the Kingdom's non-communicable disease-related deaths [23].

HF presents a significant public health challenge globally, with a prevalence of 23 million cases and a rising trend [24]. In Saudi Arabia, HF is increasingly concerning for the Ministry of Health. The projected prevalence of HF in Saudi Arabia is approximately 455,222 cases, with an estimated annual incidence of 32,200 cases [24].

Risk Factors of CVD in Saudi Arabia

In Saudi Arabia, several risk factors significantly contribute to the prevalence of CVDs. One prominent risk factor is hypertension, which affects a substantial portion of the population [18,25]. A cross-sectional study was conducted among 432 residents of Tabuk to assess the occurrence of risk factors associated with CVD. The results of the study revealed that 11% of the participants had hypertension. Furthermore, the study observed a significant association between hypertension and age in both males and females [26,27]. The combination of genetic factors, sedentary lifestyles, increased stress, and dietary patterns high in salt and processed foods contributes to the elevated rates of hypertension [28].

Obesity is another significant concern and a major risk factor for CVD in Saudi Arabia. A study conducted in Tabuk, Saudi Arabia, found that the prevalence of overweight and obesity among participants was 69.9% [27]. Another systematic review, which included 59 studies conducted in Saudi Arabia, reported that obesity rates were higher among married, older women. The modernization and urban development of Saudi Arabia have led to lifestyle and dietary changes, resulting in an increased prevalence of obesity. Furthermore, the adoption of Western dietary patterns, characterized by excessive calorie intake, high levels of saturated fats, and low consumption of fruits and vegetables, has contributed to the rising obesity rates [29,30]. This unhealthy diet has, in turn, contributed to weight gain, elevated cholesterol levels, and an increased risk of CVD, cancers, and diabetes [31]. Moreover, the sedentary nature of many jobs and limited opportunities for physical activity further exacerbate the problem [31-33]. 

There is a substantial body of evidence demonstrating a significant association between CVD and diabetes mellitus [34,35]. Diabetes has the potential to cause damage to both blood vessels and nerves that regulate cardiac function [36]. Consequently, this can lead to the development of atherosclerosis, characterized by the accumulation of cholesterol plaques within the arteries responsible for supplying blood to the heart and other vital organs [37]. In the event of plaque rupture, there is a likelihood of subsequent blockage of blood flow, which can precipitate serious cardiovascular events such as heart attacks, strokes, or peripheral vascular disease [37]. Furthermore, individuals with diabetes face a twofold higher risk of developing heart disease or experiencing a stroke compared to those without diabetes [38]. They also tend to develop heart disease at a younger age [38,39] and have a higher risk of HF [40].

In Saudi Arabia, tobacco use, particularly smoking, represents a significant risk factor for CVD. In 2018, the overall number of adults who smoked tobacco daily was 15.9% [41]. Several earlier surveys estimated the prevalence of smoking to be 12% [42], indicating a recent increase. Smoking damages blood vessels promotes the formation of blood clots, and increases the risk of CVD, cancers, and chronic obstructive pulmonary disease [43]. 

Addressing these risk factors requires a comprehensive approach involving public health initiatives, education, and policy changes. Promoting healthier lifestyles, encouraging regular physical activity, and improving access to nutritious foods can play a crucial role in reducing the prevalence of CVD in Saudi Arabia. Additionally, efforts to raise awareness about the dangers of smoking and provide support for smoking cessation are vital in combating this risk factor. By addressing these risk factors, Saudi Arabia can work toward reducing the burden of CVD and improving the overall health of its population. 

Burden of CVD on the Healthcare System

CVD has emerged as a significant public health issue in Saudi Arabia, with estimations indicating that it contributes to over 45% of total mortality in the country [18]. A bibliometric study has shed light on the substantial disease burden on the Saudi healthcare system associated with CVD [44]. 

Osman et al. conducted a cross-sectional study involving 205 patients diagnosed with angina at the Prince Sultan Cardiac Center in Riyadh. The study aimed to estimate the direct medical costs associated with ischemic heart disease. The patients were monitored until they were discharged, underwent coronary artery bypass graft surgery, or experienced a change in diagnosis. The study reported that the average cost for treating one patient was 40,164 Saudi Riyals (US$10,710), and in cases where the patient suffered from acute ST-elevation myocardial infarction, the treatment cost would rise to SAR 58,877 [45]. A further predictive study examining the prevalence and cost of CVD employed a burden of disease model. The study projected an increase to 479,500 citizens by the year 2035, leading to a threefold increase in the economic burden from US$3.5 billion to US$9.8 billion, accounting for the effects of population aging and growth [5]. These figures highlight the urgency of implementing effective preventive measures and targeted interventions to alleviate the economic impact of CVD on both the Saudi healthcare system and society. 

Impact of CVD on Health-Related Quality of Life

CVD significantly impacts the quality of life for those affected. Symptoms like dyspnea, fatigue, edema, difficulty sleeping, depression, and chest discomfort can hinder daily activities. Moreover, CVD is linked to high hospitalization and mortality rates, further diminishing the overall quality of life for individuals dealing with this condition [6]. A population-based cohort study, which included 4,910 Swedish individuals of both genders, concluded that an increasing number of CVD cases were associated with a poorer health-related quality of life (HRQoL) in affected individuals [46]. Another study carried out in China revealed that patients with coronary heart disease (CHD) experienced a decrease in their HRQoL. Additionally, it was observed that individuals with lower HRQoL faced a higher risk of combined CHD/cerebrovascular outcomes. In 2022, a study conducted in Saudi Arabia yielded comparable findings, indicating that patients with HF reported decreased HRQoL, especially among those with lower education levels and female participants. Additionally, the study revealed that most of the participants experienced poor HRQoL due to physical symptoms associated with HF, significantly affecting their ability to perform daily activities [47]. 

The Saudi 2030 Vision is a strategic plan developed by the Saudi government to transform and enhance numerous aspects of the country's economy and society. One of the primary objectives of the vision is to improve the quality of life for Saudi citizens. To achieve this, the vision focuses on promoting public health and disease prevention, investing in healthcare, advancing education and skills development, and ensuring environmental sustainability [48,49]. By implementing these initiatives, the Saudi Arabian government aims to create a more vibrant, inclusive, and prosperous society that provides its citizens, as well as residents, with better opportunities, well-being, and an overall improved quality of life [50]. However, with the increased rates of CVD in Saudi Arabia, improving the quality of life may pose a greater challenge. 

CR programs

The CR program is a comprehensive approach consisting of exercises and education [51]. These programs are designed for patients following hospital admission for various cardiac diagnoses, including myocardial infarction, acute coronary syndrome, cardiac surgery, angioplasty, defibrillator implantation, or cardiac transplantation. Additionally, individuals may also participate in these programs after experiencing any significant change in their cardiac condition, such as new-onset angina or a HF diagnosis [52]. Its primary objectives are to help patients recover as quickly and completely as possible and to minimize the risk of cardiac illness recurrence [52]. With over 50 years of history, this program boasts a substantial body of evidence supporting its efficacy, making it one of the most advantageous and cost-effective treatments accessible to patients with coronary disease [53]. 

CR programs typically consist of four phases in the UK and most of Europe. In the UK, while each NHS hospital may have a slightly different program structure, they all share common main elements in each phase of the CR program. 

In Phase 1, once patients are stable and before discharge, they receive health education regarding the causes of their condition and are advised to follow a healthy lifestyle, including physical activity, smoking cessation, and dietary changes [54]. Moreover, the CR team ensures that medications have been prescribed and that the patients are enrolled in the CR program [52]. 

During Phase 2, which takes place at the patient's home, they receive health education with reinforced advice on healthy living and are encouraged to increase their physical activities. Support from the CR team is either provided through inviting the patients to attend weekly education/health promotion classes or via telephone calls [55]. 

When it comes to Phase 3, there are two models: a hospital-based program and a community-based program [56]. This phase is the core of the CR program, and most studies are conducted to evaluate the efficacy of such programs. In Phase 3, patients are individually assessed and placed in the appropriate class for a certain period or a certain number of classes [55]. The central focus of the program is graduated exercise training, which is complemented by education. It involves a combination of exercises designed to enhance cardiovascular fitness, comprehensive education and information, relaxation techniques, and support [52]. In cases where the patient cannot attend the sessions due to their medical condition or lack of transportation, the hospital offers them a home-based program that is individually designed. Following a mutually agreed-upon period, the patient revisits the hospital for a reassessment [55]. The duration of Phase 3 varies according to the patient's risk level and responses, ranging from 4 weeks for young, fit individuals to 6 months or more for older, less fit, and higher-risk patients [52]. 

Phase 4 is the final phase, intended for the lifelong continuation of the newly adopted lifestyle habits. The British Association for CR programs has established a scheme to train instructors working in fitness centers to provide ongoing exercise support for cardiac patients. Medical follow-up is conducted in primary care, typically on an annual basis [56].

Evidence for the effectiveness of CR

More than two centuries ago, the significance of physical activity for patients with heart disease was recognized. In the early 1950s, a study conducted by Saltin and his colleagues allowed patients to walk for 3 to 5 minutes during a four-week period after cardiac events. Later, in 1968, Saltin's Dallas Bed Rest and Exercise Study provided compelling evidence of the significance of exercise and the adverse effects of prolonged bed rest following cardiac events. The study highlighted that early ambulation could prevent many complications associated with bed rest. Moreover, as far back as 1772, a physician named Heberden reported a case where he prescribed a six-month exercise regimen consisting of 30 minutes of daily sawing activity for a male patient diagnosed with a chest disorder [57]. The physiological basis of exercise benefits was discovered by a group of researchers in the late 20th century, which paved the way for the development of comprehensive CR programs aimed at assisting cardiovascular patients in their recovery and optimizing both their physical and mental well-being [57]. The efficacy of the CR program has been thoroughly validated over time, leading to strong recommendations for its implementation by numerous cardiovascular professional societies.

CR Programs and Reduced Mortality Rates

One of the most crucial measures of a CR program's success is its impact on reducing mortality rates among patients with CVDs. Graham et al. (2019) conducted a study that yielded significant insights into the effects of CR on mortality and hospital readmissions. The CR group demonstrated a statistically significant independent association with reduced all-cause mortality, with an odds ratio of 0.22. Furthermore, the CR group showed a decreased likelihood of hospital readmissions, with an odds ratio of 0.48 [58]. A further retrospective study was performed on a cohort of 2,395 patients who had undergone percutaneous coronary intervention. According to the study findings, patients who participated in CR after percutaneous coronary intervention experienced a significant 45% to 47% reduction in all-cause mortality compared to those who did not participate in CR [59]. 

Regarding elderly patients, Bradley and his colleagues studied the impact of attending CR Exercise Training (CRET) sessions on 30,161 elderly patients. The researchers compared the outcomes of patients who attended different numbers of CRET sessions. Specifically, they looked at three groups of patients: those who attended 36 sessions, those who attended 24 or fewer sessions, and those who attended 12 or fewer sessions. The research revealed a significant dose-response relationship between the number of CRET sessions attended and long-term outcomes. Patients who participated in 36 sessions, the maximum allowed, experienced a 14% lower risk of mortality compared to those who attended 24 or fewer sessions [60].

Improving Cardiovascular Risk Factors

CR programs are specifically designed interventions aimed at enhancing cardiovascular health and mitigating the potential occurrence of future cardiac complications. Evidently, these programs have demonstrated efficacy in ameliorating various cardiovascular risk factors, including blood pressure, cholesterol profiles, and body weight [61,62]. A study revealed that attending exercise-based CR led to significantly improved cardiovascular risk factors compared to non-attenders. This included higher rates of smoking cessation, increased physical activity, and slightly larger reductions in triglycerides for both genders. Moreover, men experienced less weight gain, while women demonstrated greater improvements in total cholesterol and LDL-C levels compared to non-attenders [61]. Another study at King Faisal Specialist Hospital and Research Centre in Jeddah examined how an eight-week hospital-based CR program (Phase 3) affected hemodynamic responses in post-coronary artery bypass grafting (CABG) surgery patients. The exercise training resulted in notable reductions in resting systolic and diastolic blood pressure, along with increases in post-exercise systolic blood pressure, peak heart rate, heart rate recovery, and rate-pressure product. These findings emphasize the effectiveness of supervised exercise training in enhancing hemodynamic responses and functional exercise capacity for CABG patients, highlighting the significance of implementing CR more frequently in the region [63].

Psychological Well-Being and Quality of Life

CR programs extend beyond physical health benefits; they also address the psychological and emotional aspects of CVD management. Depression, anxiety, and fear of future cardiac events are common psychological challenges faced by heart patients [64,65]. A prospective cohort study demonstrated that engaging in CR led to noteworthy enhancements in mental and psychosocial health, along with improvements in health-related quality of life [66]. Similarly, another study highlighted the promising role of CR as a multidisciplinary approach in reducing depression and anxiety while enhancing overall quality of life [67]. In a 16-week randomized controlled trial, researchers aimed to evaluate the efficacy of an aerobic exercise program compared to standard medication (sertraline hydrochloride) in treating major depressive disorder (MDD) in older patients (aged 50 years). A total of 156 participants were randomly assigned to either aerobic exercise, medication, or a combination of the two. The study found that all groups reported significant decreases in HAMD and BDI scores, with medication providing the quickest initial response. However, after 16 weeks, the exercise program revealed equivalent efficacy in lowering depression among MDD patients, suggesting that it should be considered as an alternative to antidepressants for older people with depression [68]. According to a review published in the Nature Journal, CR programs have been shown to yield favorable outcomes in terms of psychological well-being and overall quality of life in patients with heart disease [11]

Status of CR in Saudi Arabia

Characteristics of Existing CR Programs

In Saudi Arabia, while CR programs are not entirely new concepts, it is important to note that there are no posted programs on any hospital websites or even on the Ministry of Health or Saudi Heart Association websites. However, the model of a CR program at King Abdulaziz Cardiac Center, King Abdulaziz Medical City, can serve as an example of CR programs in Saudi Arabia.

At King Abdulaziz Cardiac Center, the CR program is led by nurses and supervised by physicians who work collaboratively to provide direct patient care. They develop and regularly update evidence-based protocols and algorithms for the program. The nurses are certified in DM (diabetes mellitus) management, which enables them to deliver comprehensive care while utilizing electronic charting systems in the clinics.

The CR program at King Abdulaziz Cardiac Center is staffed with a dedicated team, including full-time cardiologists, nurse specialists, nurse specialist supervisors, cardiac diabetic nurses, and prosthetic valve anticoagulation nurses. The nurse specialist ensures timely assessment of all patients admitted by the physician, meeting with them within 24 hours. During medical rounds, the nurse specialist actively contributes by providing guideline-based recommendations for patient management, coordinating patient education, and preparing for discharge.

In the outpatient clinic, patient visits are particularly intensive during the initial three months to optimize medical therapy and provide comprehensive education. Subsequently, patients are scheduled for examinations at least biannually or as needed when symptoms worsen, requiring increased medical attention. The nurse offers individualized education sessions to both patients and their family members through various means, including face-to-face interactions, phone consultations, and written materials [69].

Challenges and Barriers to Implementing CR Programs in Saudi Arabia

Like many developing countries, CR programs in Saudi Arabia can be costly [57]. Bdeir and his colleagues reported in their study that due to the high cost of CR programs, hospital facilities struggle to increase their capacity to accommodate more patients. Additionally, there is a shortage of highly qualified healthcare workers [69]. In Saudi Arabia, a significant disparity exists between CR scientific evidence and the practical implementation of programs. Furthermore, Saudi Arabia lacks a locally defined accreditation program focused on CVD management. As a result, European-based methods are widely adopted, emphasizing in-hospital care without much adaptation to the unique Saudi context [57]. Another prominent challenge lies in a considerable proportion of patients who do not initiate CR programs immediately after discharge due to the shortage of CR programs in Saudi Arabia [57]. 

A 2022 study in Saudi Arabia surveyed physicians, including general practitioners and cardiac specialists, on cardiopulmonary CR for HF patients. The results showed many physicians referred patients to CR, with general practitioners favoring hospital programs and cardiac specialists preferring home-based ones. "Fatigue related to disease" was a key factor in patient referrals, and the lack of CR centers was a significant barrier [70]. Moreover, there are limited active and fully compliant community-based CR programs in the country. According to a recent review, Saudi Arabia currently has only four established and active CR programs. Three of these programs are in Riyadh (King Faisal Specialist Hospital Research Center (KFSHRC), King Khalid University Hospital (KKUH), and King Abdulaziz University Hospital (KAAUH)), while one is situated in the Eastern province of Al-Ahssa (Prince Sultan Cardiac Centre (PSCC)). The review highlights that these programs face challenges related to staff qualifications, limited capacity, and communication gaps between the rehabilitation staff and other departments [71]. 

The availability of CR programs in Saudi Arabia is limited, with only a handful of established centers across the country. These programs face challenges related to accessibility, capacity, and qualified staff. In this study, the authors reached out to both private and government hospitals in Makkah Province, Riyadh Province, and Madinah Al Munawwarah Province to investigate the availability of CR programs. Our findings revealed that none of the private hospitals offered CR programs. Among the government hospitals, only five provided CR programs: King Abdullah Medical City in Makkah, King Fahad Hospital in Madinah (in addition to KFSHRC, KKUH, and KAAUH), and two centers, Saud Al Babtain Cardiac Center in Dammam and PSCC, offered CR services.

Furthermore, CR programs worldwide encounter a plethora of challenges. According to a recent review, CR remains significantly underutilized globally, primarily due to a multitude of disease-related and multi-level factors [51]. The review identifies three levels of challenges as follows: Health system level: One key challenge is the lack of available CR programs within healthcare systems. This shortage limits the accessibility of such programs for many patients in need, provider level: Another contributing factor is the low referral rates by physicians. Some healthcare providers might not recommend CR as part of the treatment plan, leading to missed opportunities for patients who could benefit from it [51,72], and patient-level factors: Several patient-level factors play a major role in accessing CR programs. These include transportation issues, long distances to CR facilities, financial constraints, competing responsibilities, lack of awareness about CR, and the presence of other health conditions that divert attention from the importance of CR [51].

Moreover, certain demographic groups face additional barriers to accessing CR. Women, ethnocultural minorities, older patients, individuals with lower socioeconomic status and comorbidities, and those residing in rural areas are less likely to access CR, despite often being the ones who would benefit the most from these programs [51]. Addressing these challenges is crucial to ensure that more individuals can benefit from CR and improve their overall health outcomes.

Recommendations

Initially, it is important to address the issue of low patient referrals, which can be attributed to a multitude of factors. Among these factors, one of the most prevalent is the choice of medical specialists for referral. Specifically, it has been observed that cardiologists are more inclined to refer patients to CR programs compared to family physicians [72]. To enhance the referral rate and ensure a more inclusive approach, it is recommended to implement a standardized referral checklist. This checklist can serve as a guide for all medical professionals, including family physicians and other relevant specialists, assisting them in making informed decisions regarding patient referrals to CR programs. Furthermore, one of the barriers reported by Aldhahir et al. is the availability of CR centers. Therefore, private hospitals can establish CR programs to increase availability [70].

Although the holistic approach of comprehensive programs encompasses multiple aspects of patient recovery, contributing to better outcomes and overall well-being, various research studies suggest that comprehensive CR programs yield similar effectiveness compared to exercise-based programs focused on a single phase, such as phase 3 [73,74].

Furthermore, facilitating the referral process involves providing comprehensive information to primary care centers and family physicians. A centralized list of hospitals that offer CR programs should be compiled and distributed to all primary care centers. This list should include detailed information about each CR program, enabling family physicians to make informed recommendations to their patients. By equipping family physicians with knowledge about the available CR programs, they can effectively guide and encourage patients to participate in the program that best suits their needs.

Before starting the CR program, each patient undergoing CR must undergo a thorough initial assessment to determine their specific needs and risk factors. After the assessment, personalized rehabilitation plans are created, tailored to the patient's medical history, condition, and goals [57].

Enhancing the effectiveness of CR programs and prioritizing a patient-centric approach must be done. Patient education, goal setting, and progress tracking are crucial. When patients understand the program's objectives and benefits and actively engage in setting and tracking their goals, they are more likely to stay committed and achieve better outcomes [75].

Research findings indicate that the geographical accessibility of CR programs posed a notable hurdle for patients, particularly those residing in rural areas [75,76]. To address this challenge and promote broader participation, the implementation of a home-based CR program emerges as a promising solution. This adaptation holds the potential to attract a greater number of patients to engage actively in the program.

Cardiac diseases exhibit varying factors, necessitating the adoption of a comprehensive approach through multidisciplinary collaboration. A team consisting of cardiologists, nurses, physiotherapists, nutritionists, and psychologists will offer all-encompassing care. Moreover, cooperation between hospital departments will make the program smoother and easier to handle the cardiac patients in the CR program [71].

Tailored guidelines must be developed for Arab countries that match their unique social and demographic traits. Also, a Saudi CR guideline of minimum requirements must be present for accreditation on which each institution which each cardiac institution can formulate its CRP.

The successful implementation and enhancement of CR programs in Saudi Arabia require a concerted effort from various stakeholders, including the government, the private sector (hospitals, and medical companies like Nahdi), and insurance companies. The government plays a crucial role in setting regulatory standards, allocating funds, and improving healthcare infrastructure to ensure that CR programs are accessible and of high quality. Private sector entities are instrumental in expanding the availability of CR services, delivering innovative and patient-centered care, and collaborating with other healthcare providers to offer comprehensive services. Insurance companies contribute by offering coverage or financial support (by covering part of the CR program cost) for CR programs, thereby reducing financial barriers for patient participation. these efforts can be measured by assessing patient outcomes, enrollment, and success rates in home-based CR, and increased referrals within the first year of the multidisciplinary approach.

The proposed recommendations improve the feasibility of CR programs in Saudi Arabia through several key strategies. These recommendations aim to increase patient referrals by providing a standardized referral checklist, expand CR availability by involving private hospitals, diversify CR programs to meet a broader range of patient needs, and improve information dissemination to primary care centers and family physicians. Patient empowerment through education enhances program adherence, and the introduction of home-based CR programs addresses geographical accessibility issues. Multidisciplinary care teams ensure comprehensive patient support, tailored guidelines account for local context, and stakeholder collaboration reduces financial barriers. While engaging in discussions with policymakers to ensure that our recommendations align with national public health strategies, we can enhance their impact. These measures collectively make CR programs more feasible, ultimately leading to better patient outcomes and a more accessible CR system in Saudi Arabia.

## Conclusions

Our review indicates that Saudi Arabia currently lacks a comprehensive and clear CR program for patients with heart conditions. While the government has initiated preventive services such as Anti-Smoking Clinics and Awareness Campaigns, implemented health policies like calorie menu labeling, and provided comprehensive medical services free of charge to citizens, the prevalence of CVD continues to rise. There is a pressing need for a comprehensive program that addresses and reverses the limitations experienced by patients who have endured the adverse pathophysiological and psychological consequences of cardiac events. Such a program should prioritize raising awareness among both patients and healthcare workers, promoting a healthy diet, ensuring access to gyms or fitness facilities, and providing continuous follow-up to ensure patient adherence to the plan and address their questions and concerns.

The limitations of this review are attributed to the limited data available on CVD and CR programs in Saudi Arabia. Consequently, there is a pressing need for additional studies focusing on the prevalence and incidence of CVD. Furthermore, more research is required to evaluate the effectiveness and accessibility of the ongoing CR programs.
